# Supplementary material for: From sequence to enzyme mechanism using multi-label machine learning
Source: BMC Bioinformatics. 2014 May 19;15:150. doi: 10.1186/1471-2105-15-150 (PMC4229970; doi:10.1186/1471-2105-15-150)
Supplement: Additional file 2 — Java code of ml2db. Additional file ml2db_code.tar.gz contains the Java source code to run the multi-label machine learning experiments and save the results to database. The code’s Javadoc is included. [file 1471-2105-15-150-S2.zip › additional file 2/ml2db/utils/doc/uk/ac/standrews/utils/main/database/DbManager.html]

DbManager


JavaScript is disabled on your browser.


- Overview
- Package
- Class
- Use
- Tree
- Deprecated
- Index
- Help

- Prev Class
- Next Class

- Frames
- No Frames

- All Classes

- Summary:
- Nested |
- Field |
- Constr |
- Method

- Detail:
- Field |
- Constr |
- Method


uk.ac.standrews.utils.main.database

## Class DbManager

- java.lang.Object
- - uk.ac.standrews.utils.main.database.Manager
  - - uk.ac.standrews.utils.main.database.DbManager

- All Implemented Interfaces:
  :   java.io.Serializable

  Direct Known Subclasses:
  :   TableManager

  ---

    

  ```
  public class DbManager
  extends Manager
  implements java.io.Serializable
  ```

  A database manager provides object to create a database, write and read from
  it. A manager manages the trio: `DbCreator` to create the tables,
  `DbReader` to read data from and `DbWriter` to write data to the
  database.

  Version:
  :   20 Feb 2009

  Author:
  :   Luna De Ferrari luna.deferrari-at-ed.ac.uk

  See Also:
  :   Serialized Form

- - ### Field Summary

    Fields

    | Modifier and Type | Field and Description |
    | --- | --- |
    | `DbWriter` | `m_dbWriter` the database recorder |
  - ### Constructor Summary

    Constructors

    Constructor and Description

    `DbManager(DbManager dbManager)`

    Constructor 2: creates the database manager from another one

    `DbManager(java.lang.String dbConnPath)`

    Constructor 1: creates the database connection and the classes for
    reading/writing to database from database properties file
  - ### Method Summary

    Methods

    | Modifier and Type | Method and Description |
    | --- | --- |
    | `void` | `closeConnection()` |
    | `boolean` | `connectionIsValid()` |
    | `java.lang.String` | `getDatabaseName()` |
    | `DbConn` | `getDbConnection()` |
    | `DbCreator` | `getDbCreator()` |
    | `DbReader` | `getDbReader()` |
    | `DbWriter` | `getDbWriter()` |
    | `void` | `refreshConnection()` |
    | `void` | `runAnalyzeTable()` Runs the 'ANALYZE TABLE tablename' SQL command for all the tables in a mysql database. |

    - ### Methods inherited from class java.lang.Object

      `equals, getClass, hashCode, notify, notifyAll, toString, wait, wait, wait`

- - ### Field Detail


    - #### m\_dbWriter

      ```
      public DbWriter m_dbWriter
      ```

      the database recorder
  - ### Constructor Detail


    - #### DbManager

      ```
      public DbManager(DbManager dbManager)
      ```

      Constructor 2: creates the database manager from another one


    - #### DbManager

      ```
      public DbManager(java.lang.String dbConnPath)
      ```

      Constructor 1: creates the database connection and the classes for
      reading/writing to database from database properties file
  - ### Method Detail


    - #### closeConnection

      ```
      public void closeConnection()
      ```


    - #### connectionIsValid

      ```
      public boolean connectionIsValid()
      ```


    - #### getDatabaseName

      ```
      public java.lang.String getDatabaseName()
      ```


    - #### getDbConnection

      ```
      public DbConn getDbConnection()
      ```


    - #### getDbCreator

      ```
      public DbCreator getDbCreator()
      ```


    - #### getDbReader

      ```
      public DbReader getDbReader()
      ```


    - #### getDbWriter

      ```
      public DbWriter getDbWriter()
      ```


    - #### refreshConnection

      ```
      public void refreshConnection()
      ```


    - #### runAnalyzeTable

      ```
      public void runAnalyzeTable()
      ```

      Runs the 'ANALYZE TABLE tablename' SQL command for all the tables in a
      mysql database. (Refresh indexes etc.) Good for performance.


- Overview
- Package
- Class
- Use
- Tree
- Deprecated
- Index
- Help

- Prev Class
- Next Class

- Frames
- No Frames

- All Classes

- Summary:
- Nested |
- Field |
- Constr |
- Method

- Detail:
- Field |
- Constr |
- Method
